# Plasma Endocannabinoid Alterations in Individuals with Substance Use Disorder are Dependent on the “Mirror Effect” of Schizophrenia

**DOI:** 10.3389/fpsyt.2012.00085

**Published:** 2012-09-25

**Authors:** Joëlle Desfossés, Emmanuel Stip, Lahcen Ait Bentaleb, Olivier Lipp, Jean-Pierre Chiasson, Alexandra Furtos, Karine Venne, Edouard Kouassi, Stéphane Potvin

**Affiliations:** ^1^Department of Psychiatry, Faculty of Medicine, Fernand-Seguin Research Centre, Université de MontréalMontréal, QC, Canada; ^2^Department of Psychiatry, Faculty of Medicine, Centre Hospitalier de l’Université de Montréal, Université de MontréalMontréal, QC, Canada; ^3^Clinique du Nouveau-DépartMontréal, QC, Canada; ^4^Department of Chemistry, University of MontrealMontréal, QC, Canada; ^5^Department of Medicine, Faculty of Medicine, Maisonneuve-Rosemont Hospital Research Center, Université de MontréalMontréal, QC, Canada

**Keywords:** schizophrenia, endocannabinoids, anandamide, oleoylethanolamide, substance use disorder, vulnerability marker

## Abstract

Schizophrenia is a complex psychiatric disorder strongly associated with substance use disorders. Theoretically, schizophrenia and SUD may share endocannabinoid alterations in the brain reward system. The main endocannabinoids, anandamide, and 2-arachidonoylglycerol, are lipids which bind cannabinoid receptors. Oleoylethanolamide (OEA), a fatty-acid ethanolamide, binds peroxisome proliferator-activated receptors. The endocannabinoid system has been shown to be impaired in schizophrenia, and recently, our group has shown that schizophrenia patients with SUD have elevated peripheral levels of anandamide and OEA that do not normalize after 3-month treatment with quetiapine. Objective For comparative purposes, we aimed to measure endocannabinoids in non-psychosis substance abusers and non-abusing schizophrenia patients. Methods Using liquid chromatography and mass spectrometry, we measured plasma levels of anandamide and OEA in non-psychosis SUD patients, non-abusing schizophrenia patients, and healthy controls. In an open-label manner, all patients received 12-week treatment with quetiapine. Results Anandamide and OEA were reduced in substance abusers without schizophrenia, relative to healthy controls (*p* < 0.05). Both endocannabinoids were unchanged in non-abusing schizophrenia patients. After quetiapine, anandamide, and OEA levels remained significantly reduced the SUD group (*p* < 0.05). Discussion Taken together with results of our previous study performed in dual-diagnosis patients, our results suggest that peripheral anandamide and OEA levels are impaired in patients with SUD in opposite ways according to the presence or absence of schizophrenia. Endocannabinoid alterations did not change with treatment, suggesting that they are trait markers. Further studies are necessary to understand the role of endocannabinoids in substance abusers with and without schizophrenia and to examine therapeutic implications.

## Introduction

Schizophrenia is a complex disease encompassing 0.87% of the general population (Perala et al., 2007). Psychiatric symptoms and comorbidities such as substance use disorder (SUD) contribute to the associated disability. The lifetime prevalence of SUD is approximately 40% in schizophrenia (Regier et al., 1990). It has a negative impact on drug compliance, psychotic relapses, and on prognosis. Patients with schizophrenia are more prone to substance abuse than the general population and 25% have a lifetime prevalence of cannabis abuse/dependence, the most widely used illicit psychoactive substance worldwide (Jablensky, 2000). Cannabinoid intoxication can provoke cognitive deficits and psychiatric symptoms akin to the cognitive and positive symptoms of schizophrenia (D’Souza et al., 2004). Chronically, cannabis smoking seems to provoke an amotivational syndrome similar to the negative symptoms of the disorder (Nunez and Gurpegui, 2002). Cannabinoid intoxication has therefore become a valid psychosis model, because its effects are representative of the full spectrum of schizophrenia symptoms. The endogenous cannabinoid (ECB) system may help to understand the link between schizophrenia and substance abuse.

The main ECBs, anandamide, and 2-arachidonoylglycerol (2-AG), are lipids naturally derived from membrane precursors which bind cannabinoid receptors (CB_1_, CB_2_; Ameri, 1999). Although structurally related to anandamide, palmitoylethanolamide (PEA), and oleoylethanolamide (OEA) are two non-cannabinoid natural bioactive fatty-acid ethanolamides (FAEA), as these lipids do not bind cannabinoids receptors, but bind with high affinity to the peroxisome-proliferator-activated receptor- alpha (PPAR-α; Fu et al., 2003). ECBs are synthesized on request by neurotransmitters and the ECB system is involved in several neuromodulation processes.

Preliminary evidence suggests that the ECB system is disturbed in schizophrenia (Desfossés et al., 2010). Indeed, postmortem human brain studies have shown that CB_1_ receptor density is elevated in several brain regions involved in the pathophysiology of schizophrenia, such as the prefrontal cortex, the hippocampus, and the basal ganglia (Dean et al., 2001). Similarly, a recent positron emission tomography (PET) study has brought new insights about the link between schizophrenia and CB_1_ receptors. *In vivo*, it showed an elevation of CB_1_ receptor binding in patients with schizophrenia across many brain regions, significantly in the pons, relative to healthy controls (HC; Wong et al., 2010). CB_1_ receptor functioning may also be altered in schizophrenia in brain regions involved in cognition. Moreover, anandamide levels are increased in the cerebrospinal fluid (CSF) of patients with schizophrenia compared to healthy controls (HC) (Leweke et al., 1999) and to individuals with other psychiatric disorders (Giuffrida et al., 2004). Inconsistently, anandamide levels have also been shown to be elevated in the serum of schizophrenia patients (De Marchi et al., 2003), suggesting that anandamide may play a protective role in psychosis homeostasis.

Theoretically, schizophrenia and SUD may share ECB alterations in the brain reward system. The study of ECB ligands, such as anandamide and OEA, may provide mechanistic explanations for the increased vulnerability to SUD in schizophrenia patients and/or the increased vulnerability to develop psychotic symptoms in individuals with SUD. Studies are therefore needed to explore the role of the ECB system in the pathophysiology of schizophrenia which consider the impact of substance abuse.

A previous study of our group of research examined plasma endocannabinoid in patients with dual-diagnosis schizophrenia (Potvin et al., 2008). Plasma anandamide and OEA levels were elevated at baseline in patients compared to controls and were not modified after 12 weeks of treatment with the second-generation antipsychotic quetiapine. In the present study, we report plasma anandamide and OEA concentrations in substance abusers without schizophrenia and in non-abusing schizophrenia patients assessed in the same clinical conditions and included following the same criteria as the patients with dual-diagnosis. We examined endocannabinoid outcomes in substance abusers without schizophrenia and in non-abusing schizophrenia patients undergoing 12-week treatment with quetiapine. This antipsychotic was chosen because it has previously been shown to improve substance use and psychiatric outcomes in psychosis, dual-diagnosis, and non-psychosis patients (Kampman et al., 2007; Martinotti et al., 2008; Potvin et al., 2008; Rizkallah et al., 2010). Importantly, this is the first study of its kind to trace endocannabinoid outcomes prospectively in these groups. During the trial, we sought to determine whether: (i) baseline plasma anandamide and OEA concentrations were different between patients and controls, (ii) plasma anandamide and OEA levels varied from baseline to endpoint.

## Materials and Methods

### Participants

To complement the data collected previously in dual-diagnosis schizophrenia patients and HC (Potvin et al., 2008), three groups of participants were recruited, namely: (i) non-abusing patients with schizophrenia-spectrum disorders (schizophrenia, schizoaffective disorder, schizophreniform disorder; SCZ group); (ii) non-psychotic substance abusers in detoxification (SUD group); and (iii) a new group of HC. Psychiatric and SUD diagnoses were by well-trained psychiatrists and physicians, and were all based on DSM-IV criteria. SUD diagnoses were complemented with urine drug screenings. All participants signed a detailed consent form. The study was approved by the local ethics committee.

For all patient groups (SCZ and SUD), exclusion criteria were: (i) patients already on clozapine or quetiapine; (ii) patients hospitalized in a psychiatric unit; (iii) pregnancy; (iv) female subjects of childbearing potential or inadequate contraception; and (v) clinically meaningful unstable, renal, hepatic, cardiovascular, respiratory, cerebro-vascular, or other serious, progressive physical disease. For the SCZ group, patients were excluded if their total score on the Positive and Negative Syndrome Scale (PANSS; Kay et al., 1987) was lower than 65. Importantly, these exclusion criteria are the same as the ones used in our previous study involving dual-diagnosis patients (Potvin et al., 2008). SUD patients were diagnosed with one or more of the following SUD: alcohol (*n* = 24); cannabis (*n* = 20); stimulants (*n* = 15). SCZ patient received one or more first-generation (*n* = 5) or second-generation antipsychotics (*n* = 14), four patients were drug-free. For study completers, the mean quetiapine dose was 478.3 mg (272.0) and 150.0 mg (117.7) in the SCZ and SUD groups, respectively. Of note, in the previous study, dual-diagnosis schizophrenia patients used similar psychoactive substances, were taking similar types of antipsychotics and received similar doses of quetiapine (for more information, see Potvin et al., 2008).

### Clinical assessments

Psychiatric symptoms were measured using the PANSS and the Calgary Depression Scale for Schizophrenia (CDSS; Addington et al., 1993). Quantities of substances used in the last week were also registered, using the TimeLine Follow-Back (TLFB) procedure (Sobell et al., 1992). Quantities used were noted for all substances. Amount spent on substances was calculated based on the value market in Quebec province (Canada). To complement our evaluation of SUD, urine screenings were performed before and after treatment. SUD severity was also evaluated using the Alcohol Use and the Drug Use Scales (Drake et al., 1990; for more information, see Potvin et al., 2008; Rizkallah et al., 2010).

### Analysis of AEA and OEA from plasma

We collected blood samples (2 mL) using heparinized tubes. Within 1 h, blood samples were centrifuged (3200 rpm for 15 min), and plasma (1 mL) was stored at −80°C in glass vials. Blood samples were collected before and after treatment. All extraction manipulations were done on ice or at 4°C. Plasma (500 μL) was spiked with 1 ng of internal standard AEA-d8. All samples were thoroughly vortexed and centrifuged at 15,000 *g* for 5 min. The Solid Phase Extraction was conducted according to the method developed by Marczylo et al. (2009) with slight modifications. LC-MS-MS analyses were performed on a Surveyor coupled to a TSQ Quantum AM Ultra (ThermoFisher, San Jose, CA, USA) operating in positive electrospray mode. For each analyte, silver adducts were selected for multiple reaction monitoring (MRM). Injections of 5 μL were done on a 2.6 μm Kinetex C18 3.0 × 100 mm column (Phenomenex, Torrance, CA, USA) and separated using a linear gradient. The eluants consisted of 70 μM aqueous silver acetate (solvent A), 70 μM silver acetate in 90% methanol/10% H_2_O (solvent B), and 70 μM silver acetate in 90% acetonitrile/10% H_2_O (solvent C). The separation started at 30%A, 20%B, and 50%C and ended at 0%A, 20%B, and 80%C for a total run time of 20 min. The lowest limits of quantitation were 0.10 ng/mL for AEA and 0.17 ng/mL for OEA.

### Statistical analyses

Baseline differences between SCZ patients, SUD patients and HC were analyzed using one-way analyses of variance (ANOVA) with group as the independent variable. Dichotomous variables were evaluated using Pearson’s chi-square test. Multiple comparisons were performed using the Bonferroni correction. Changes in substance abuse and psychiatric symptoms were analyzed using repeated-measures ANOVA. Last-observation carried forward (LOCF) was used. The level of significance was set at *p* < 0.05. Statistical analyses were performed using the Predictive Analytics SoftWare (PASW; version 18).

## Results

### Participants

Twenty-five SCZ patients were prescribed quetiapine; of these, two were lost-to-follow-up. Thirty-eight SUD patients were recruited, and 33 SUD patients were prescribed quetiapine; of these, two were lost-to-follow-up, two dropped out due to side-effects, and three dropped out due to SUD relapse. Therefore, LOCF analysis was available for 23 and 26 patients in the SCZ and SUD groups, respectively.

### Socio-demographic variables

SCZ patients, SUD patients, and HC did not differ in terms of age [HC: 37.1 (12.5); SCZ: 40.3 (12.6); SUD: 37.9 (12.1); *F* = 0.5; *p* = 0.626], sex [HC: 16 males (M), 11 females (F); SCZ: 17M, 8F; SUD: 25M, 13F; χ^2^ = 0.5; *p* = 0.785], ethnicity (HC: 26 Caucasian; SCZ: 22 Caucasian; SUD: 34 Caucasian; χ^2^ = 1.3; *p* = 0.517), and weight [HC: 76.3 (16.2); SCZ: 76.9 (13.2); SUD: 76.5 (18.0); *F* = 0.01; *p* = 0.992].

### SUD outcomes

In SUD patients, all substance use outcomes significantly improved during treatment (*p* < 0.05; for more information, see Rizkallah et al., 2010).

### Psychiatric symptoms

On baseline, SCZ and SUD patients had similar positive symptoms [SCZ: 17.4 (4.4); SUD: 16.2 (5.1); *F* = 3.2; *p* = 0.08]; SCZ patients had more negative symptoms than SUD patients [SCZ: 16.9 (4.7); SUD: 13.6 (4.7); *F* = 7.2; *p* = 0.01]; and SUD patients had more depressive symptoms than SCZ patients [SCZ: 3.6 (4.4); SUD: 6.8 (4.4); *F* = 8.4; *p* = 0.005; Zhornitsky et al., 2011]. In SCZ and SUD patients, psychiatric symptoms (positive, negative, and depressive) significantly improved during treatment (*p* < 0.05; for more information, see Zhornitsky et al., 2011).

### Endocannabinoids

Compared to HC, plasma anandamide, and OEA levels were significantly reduced in SUD patients, but not in SCZ patients (Table 1). Peripheral anandamide and OEA remained reduced in the SUD group after quetiapine treatment (Table 2). Noteworthy, sub-analyses targeting specific psychoactive substances (alcohol, cannabis, or stimulants) revealed that AEA and OEA levels were reduced in the SUD group, compared to HC, irrespective of the substance (*p* < 0.05).

**Table 1:** **Anandamide and OEA at baseline according to schizophrenia or substance use disorder compared to healthy controls**.

Variable	HC (*n* = 27)	SCZ patients (*n* = 25)	SUD patients (*n* = 38)	Statistics	Multiple comparison*
Anandamide (ng/mL)	1.1 (0.4)	1.1 (0.2)**	0.7 (0.2)	*F* = 24.3; *p* = 0.0001	SUD < HC and SCZ
OEA (ng/mL)	1.6 (0.7)	1.3 (0.4)**	1.0 (0.5)	*F* = 11.2; *p* = 0.0001	SUD < HC

HC, healthy controls; OEA, oleoylethanolamide; SCZ, schizophrenia; SUD, substance use disorders; *After Bonferroni correction; **n = 21, missing data.

**Table 2:** **Changes in plasma endocannabinoid levels during quetiapine treatment**.

Variable	Baseline	LOCF data	Statistics
Non-abusing schizophrenia patients (*n* = 15)*			
Anandamide (ng/mL)	1.1 (0.3)	1.0 (0.3)	*F* = 0.7; *p* = 0.415
Oleoylethanolamide (ng/mL)	1.3 (0.4)	1.3 (0.3)	*F* = 0.2; *p* = 0.635
Non-psychosis patients with substance use disorders (*n* = 24)			
Anandamide (ng/mL)	0.7 (0.2)	0.7 (0.3)	*F* = 0.3; *p* = 0.599
Oleoylethanolamide (ng/mL)	1.0 (0.5)	1.1 (0.6)	*F* = 0.5; *p* = 0.508

LOCF, last-observation-carried-forward; *missing data.

## Discussion

Previously, our group has measured plasma levels of anandamide and OEA in 29 schizophrenia patients with comorbid SUDs (cannabis > alcohol > cocaine), before and after 12-week treatment with quetiapine. We found that peripheral levels of anandamide and OEA were both increased in dual-diagnosis patients, relative to controls. Noteworthy, these elevations were not normalized by quetiapine treatment, despite improvements psychiatric and SUD outcomes (Potvin et al., 2008). In the current study, plasma anandamide levels were reduced in SUD patients, but normal in non-abusing schizophrenia patients, and both anandamide and OEA levels remained reduced in the SUD group after quetiapine treatment. Taken together, the results from both studies (previous and current) highlight a striking opposite phenomenon, namely that peripheral anandamide and OEA levels are elevated in dual-diagnosis schizophrenia while both being reduced in non-psychosis substance abusers. Given that anandamide and OEA alterations were not normalized during treatment, such results strongly suggest that ECB alterations are trait markers of SUD.

Our results have shown that the involvement of ECBs in SUDs is expressed in opposite ways, according to the presence or absence of a comorbid schizophrenia diagnosis, suggesting a “mirror effect.” Such a result must be interpreted cautiously. On one hand, given the role of ECBs in the brain reward system, it is possible that elevations of anandamide and OEA levels constitute risk factors leading to SUDs in individuals with psychosis vulnerability. Alternatively, it is also possible that anandamide and OEA elevations are endogenous markers of psychosis vulnerability in the context of substance abuse, more precisely cannabis abuse. Consistently with this latter interpretation, it has been shown that cannabis smoking can produce psychotomimetic effects, and that it moderately increases the risk for psychotic symptoms (Arseneault et al., 2004).

To explain the striking “mirror effect” reported here, one potential explanation may be related to the fact that Δ9-THC, the main psychoactive agent of cannabis, is a partial agonist at CB_1_ receptors (Pertwee, 2005), and may therefore produce opposing effects on anandamide and OEA based on *a priori* psychosis vulnerability. Alternatively, it may produce different psychiatric effects based on the individual’s basal ECB tone. To explain this “mirror effect,” it may also be relevant to consider the balance between dopamine and ECBs in psychosis homeostasis. Using PET, various studies have shown that amphetamine-induced dopamine release is increased in schizophrenia, mostly in the acute phase of illness (Laruelle, 1998; Guillin et al., 2007). On the opposite, the chronic use of psychoactive substances has been shown to down-regulate striatal dopaminergic neurotransmission (Volkow et al., 1993, 1996, 2001; Hietala et al., 1994; Wang et al., 1997; Ginovart et al., 1999; Martinez et al., 2004; Lee et al., 2009). Given that anandamide has been shown to inhibit dopamine release in the striatum, as a retrograde messenger (Giuffrida et al., 1999), and that OEA inhibits drug-induced dopamine elevations in the reward system via PPAR-α (Melis et al., 2008), one may hypothesize that the inverted endocannabinoid alterations in dual-diagnosis patients and SUD patients are the results of complex interactions with dopamine. Against this view, however, it is important to mention that the dopaminergic dysfunctions associated with both schizophrenia and substance abuse are mostly transient (Laruelle, 1998; Volkow et al., 2001), whereas the endocannabinoid alterations reported here and in our previous study are persistent in time (Potvin et al., 2008).

One of the research implications of our results is that longitudinal studies would be needed in adolescent substance users, before they develop schizophrenia or SUD, to understand the involvement of ECBs in SUD – with a particular attention to specific substances, such as alcohol, cannabis, and stimulants. Our results may also have future implications for the pharmacological treatment of SUDs, as they suggest that CB_1_ or PPARs agonists would be required in substance abusers, whereas CB_1_ or PPAR antagonists would be required in dual-diagnosis schizophrenia patients. Although CB_1_ antagonists have shown promise in pre-clinical models of addiction to various substances (Economidou et al., 2006; Femenia et al., 2010; Yu et al., 2011), and PPAR-γ agonists have been shown to reduce alcohol drinking in rodents (Stopponi et al., 2011), the clinical efficacy of CB_1_ antagonists for SUD remains to be proven (Cahill and Ussher, 2011) and PPAR agonists have not been tested thus far in humans. The adequate pharmacological modulation of ECB tone could also provide therapeutic targets. One of them is fatty-acid amide hydrolase (FAAH), the enzyme responsible for the degradation of anandamide and OEA, which offers a more selective way to alter ECB activity. FAAH inhibitors seem to be potent modulators of motivation and goal-directed behaviors associated with SUD (such as cocaine) without reducing consumption in rat models (Adamczyk et al., 2009). However, FAAH inhibitors have not been tested thus far for the treatment of alcohol or drug addiction in humans.

Our study has three main limitations. First, our study was limited by the inclusion of various psychoactive substances, making the attribution of our results to specific substances difficult. Another limitation of our study is the inclusion in the non-abusing schizophrenia group having a small number of patients. The lack of difference in ECB levels between non-abusing schizophrenia patients and controls could therefore be explained by a type-II error. Finally, we measured peripheral, not central, levels of anandamide, and OEA, and this may have influenced our results. For instance, the absence of difference in plasma anandamide and OEA between non-abusing patients with schizophrenia-spectrum disorders and controls might be explained by various peripheral influences, especially since ECBs are involved in various systems (Hohmann and Suplita, 2006; Pacher et al., 2006; Matias and Di Marzo, 2007; Hiley, 2009). Moreover, the absence of ECB alterations in the schizophrenia group does not preclude central ECB disturbances. Thus far, two studies have shown that anandamide levels are elevated in the CSF of schizophrenia patients (Leweke et al., 1999; Giuffrida et al., 2004) and another one showed that CSF anandamide levels are also elevated in the prodromal phase of psychosis (Koethe et al., 2009). Importantly, CSF OEA levels were not altered in these three studies. As for the peripheral measurement of ECBs in schizophrenia, De Marchi et al. (2003) showed that blood anandamide levels were elevated in small group (*n* = 12) of schizophrenia patients, during the acute phase of illness. In contrast, the serum levels of both anandamide and OEA were shown to be unaltered in schizophrenia (Giuffrida et al., 2004) as well as in the prodromal phase of psychosis (Koethe et al., 2009).

## Conclusion

Our study has highlighted a “mirror effect” of comorbid schizophrenia on peripheral ECB alterations in SUDs. Given the lack of changes during treatment in ECB levels, our results further suggest that ECB alterations in SUD are trait-, not state-, dependent. Longitudinal studies are required to investigate the biological reasons for the inverted pattern of ECB alterations in substance abusers with and without schizophrenia. The precise role of specific psychoactive substances on our results will also need to be determined. Research in this field could significantly help elucidate the neurobiological dysfunctions underlying the schizophrenia-substance abuse comorbidity, and it may also open new therapeutic perspectives in the future.

## Conflict of Interest Statement

The authors declare that the research was conducted in the absence of any commercial or financial relationships that could be construed as a potential conflict of interest.
